# Inconsistent condom use among HIV-positive women in the “Treatment as Prevention Era”: data from the Italian DIDI study

**DOI:** 10.7448/IAS.16.1.18591

**Published:** 2013-10-16

**Authors:** Paola Cicconi, Antonella d'Arminio Monforte, Antonella Castagna, Tiziana Quirino, Anna Alessandrini, Miriam Gargiulo, Daniela Francisci, Enza Anzalone, Giuseppina Liuzzi, Paola Pierro, Adriana Ammassari

**Affiliations:** 1 Department of Health Sciences, Institute of Infectious and Tropical Diseases, San Paolo University Hospital, Milan, Italy; 2 Department of Infectious Diseases, San Raffaele Scientific Institute, Milan, Italy; 3 Department of Infectious Diseases, Busto Arsizio Hospital, Busto Arsizio, Italy; 4 Clinic of Infectious Diseases, University of Genoa, San Martino Hospital, Italy; 5 Third Division of Infection Diseases, AORN dei Colli, Naples, Italy; 6 Department of Experimental Medicine and Biochemical Sciences, University of Perugia, Italy; 7 Department of Infectious Diseases, Hospital of Frosinone, Frosinone, Italy; 8 Department of Infectious Diseases, Istituto Nazionale di Malattie Infettive, L. Spallanzani, Rome; * See Appendix

**Keywords:** HIV, women, condom use, antiviral therapy, adherence

## Abstract

**Introduction:**

Translation of the evidence regarding the protective role of highly active antiretroviral therapy (HAART) on HIV sexual transmission rates into sexual behaviour patterns of HIV-infected subjects remains largely unexplored. This study aims to describe frequency of self-reported condom use among women living with HIV in Italy and to investigate the variables associated with inconsistent condom use (ICU).

**Methods:**

DIDI (*D*onne con *I*nfezione *D*a H*I*V) is an Italian multicentre study based on a questionnaire survey performed during November 2010 and February 2011. Women-reported frequency of condom use was dichotomized in “always” versus “at times”/“never” (ICU).

**Results:**

Among 343 women, prevalence of ICU was 44.3%. Women declared a stable partnership with an HIV-negative (38%) and with an HIV-positive person (43%), or an occasional sexual partner (19%). Among the 194 women engaged in a stable HIV-negative or an occasional partnership, 51% reported fear of infecting the partner. Nonetheless, 43% did not disclose HIV-positive status. Less than 5% of women used contraceptive methods other than condoms. At multivariable analysis, variables associated with ICU in the subgroup of women with a stable HIV-negative or an occasional HIV-unknown partner were: having an occasional partner (AOR 3.51, 95% confidence interval [CI] 1.44–8.54, *p*=0.005), and reporting fear of infecting the sexual partner (AOR 3.20, 95% CI 1.43–7.16, *p*=0.004). Current use of HAART together with virological control in plasma level did not predict ICU after adjusting for demographic, behavioural and HIV-related factors. With regard to socio-demographic factors, lower education was the only variable significantly associated with ICU in the multivariate analysis (AOR 2.27, 95% CI 1.07–4.82, *p*=0.03). No association was found between high adherence to HAART and ICU after adjusting for potential confounders (AOR 0.89, 95% CI 0.39–2.01, *p*=0.78).

**Conclusions:**

Currently in Italy, the use of HAART with undetectable HIV RNA in plasma as well as antiretroviral adherence is not associated with a specific condom use pattern in women living with HIV and engaged with a sero-discordant or an HIV-unknown partner. This might suggest that the awareness of the protective role of antiretroviral treatment on HIV sexual transmission is still limited among HIV-infected persons, at least in this country.

## Introduction

Intense interest has developed in the use of highly active antiretroviral therapy (HAART) to prevent HIV transmission. Indeed, the belief in the power of HIV suppression to stop secondary transmission is so strong, that the Swiss AIDS Commission issued a declaration indicating that, under selected circumstances, HIV status-discordant couples might engage in unprotected sexual intercourse with minimal risk [1]. More recently, the protective effect of treatment on HIV-1 sexual transmission has been confirmed by scientific evidence and a meta-analysis [2,3]. The potential changes in sexual risk behaviours consequent to the patients’ awareness of this information are still a subject of interest in an on-going debate. A recent study, did not report a substantial change in the quality of sexual life, HAART adherence and condom use in people living with HIV, aware of the “Swiss Statement” [4]. Similarly, no evidence of sexual behavioural risk compensation was observed among injection drug users following initiation of HAART [5]. However, among men who have sex with men receiving HAART, self-reported undetectable HIV plasma viraemia resulted significantly associated with unprotected sex with HIV-discordant partners, suggesting that perceived viral load status might influence sexual behaviour and condom use in this population [6]. Focusing on women, explicit or latent motherhood desire as well as gender power inequalities shall be added to the factors that influence sexual behaviour, as well as condom and contraceptive use. Several studies on women with HIV infection detected high rates of unexpected pregnancies [7,8]. Other authors found evidence of difficulty in women at negotiating condom use with their male partners, in part due to HIV disclosure [9–13].

In the current “Treatment as Prevention Era”, this study aims to describe the frequency of self-reported condom use among women living with HIV in Italy and to investigate the variables associated with inconsistent condom use (ICU). In particular, the possible role of HAART-associated virological control in plasma and adherence to antiretrovirals is explored.

## Methods

### The DIDI study

DIDI (**D**onne con **I**nfezione **D**a H**I**V) is an Italian multicentre study based on a questionnaire survey performed in 585 HIV-positive women during November 2010 and February 2011. The primary purpose of the study was to elicit information in order to draw a profile of HIV-positive Italian women and well-integrated female migrants living in Italy. Local human subjects committees’ of the principal investigators’ clinical centres (San Paolo University Hospital, Milan; INMI “L. Spallanzani,” Rome) gave their approval to the study and written informed consent was obtained from study participants.

Healthcare workers of the 16 clinical centres distributed the anonymous in-depth questionnaire to all women aged 18 years or older consecutively observed at one of their routine follow-up visits. Women with insufficient knowledge of the Italian language were excluded. In order to avoid desirability bias and, moreover, to guarantee the patient's confidentiality, the women inserted the completed questionnaire in an envelope, sealed it and gave it to the study staff. The clinical staff (i.e. caring physician and nurses) had no access to information collected by the questionnaire.

Items covered by the questionnaire included the following: socio-demographic characteristics, sexual and gynaecological health, motherhood desire, HIV disclosure to sexual partner, fear of infecting the partner, physical and mental health, data on recreational drug use and smoking, spiritual and religious attitude. Questions were mostly structured as closed-ended, with ordered response choices.

In particular, women were asked to report frequency of condom use (“always,” “at times,” “never”), use and type of other contraceptives, type of partnership (“stable,” “occasional”), and in case of a stable partner, the partner's HIV serostatus (“positive,” “negative,” “unknown”). HAART adherence was investigated by three questions: “How many times did you take your antiretroviral medications in the last month?” (visual analogue scale [VAS] from 0=never to 100%=always); “Did you forget to take your medications in the last week?” (“yes,” “no”); “In the last three months, did you run out of medications before prescription refill?” (“yes,” “no”); “In the last three months, did it happen that you stopped medication intake for two or more days?” (“yes,” “no”).

HIV transmission mode, CDC staging, viro-immunological parameters, antiretroviral drug experience including start and stop date of each drug, co-infection with hepatitis viruses, and data on other sexually transmitted diseases were available from the patients’ records.

### Statistics

For the purpose of this study, ICU was defined based on the women self-reported variable: use of condoms “never” or “at times.” On the contrary, consistent condom was assigned to cases that declared to use condoms “always.” Women reporting ICU, were compared to those declaring condom use on a regular basis, with regard to socio-demographic, behavioural, and clinical characteristics using Chi-square and Wilcoxon tests, as appropriated. To adequately investigate HIV prevention prospective, we focused on women who reported to have a stable HIV negative or an occasional HIV unknown partner. A multivariable, logistic regression was used in order to identify independent predictors of ICU in this setting. A combined variable of interest was built based on HAART administration and plasma viral load at the time of questionnaire: (i) off-HAART; (ii) on HAART with virological control (HIV-RNA<50 cp/mL); and (iii) on HAART without virological control (HIV-RNA≥50 cp/mL). In a sensitivity analysis, the viral load threshold of 200 cp/mL was chosen because blips of viral load up to that level occur quite frequently without a consequence for antiretroviral management. Other variables included in the model were: age at enrolment, citizenship (migrant vs. native Italian), education level (primary school vs. high school/university), monthly salary (cut off at €350), menopause (≥12 months reported amenorrhoea), motherhood desire, having ≥1 child, mode of HIV transmission (intravenous drug use [IVDU] vs. sexually transmitted), years from HIV diagnosis, partner status (occasional, stable HIV negative), HIV disclosure to sexual partner (yes, no), fear to infect the partner (yes, no), number of partners in the last year (1 vs. ≥1).

We then wanted to focus on the potential association of adherence with HAART on ICU. High medication adherence was defined as reporting all of the following: VAS score ≥95%, no missed doses in the last week, no drug supply interruption, no HAART discontinuation in the last three months. Predictors of ICU were assessed by logistic regression; variables included were the same as in the main analysis.

Women who reported no sexual activity in the previous year or with missing data on the frequency of condom use were excluded from the study.

## Results

Among the 585 women enrolled in the DIDI study, 362 (62%) reported sexual activity in the previous year. Of these, 19 (5%) did not answer the question on condom use and were therefore excluded from the analysis.

Socio-demographic, behavioural and HIV-related characteristics of the 343 included women, according to frequency of condom use, are presented in Table 1. Median age was 43 years (IQR 37–47) and migrant status accounted for 13%. Overall, interviewed women had a long history of known HIV infection (median years 12 [IQR 6–19]), 20% were classified as CDC stage C, 311 (91%) were receiving HAART, and 270 (79%) had virological control in plasma. Menopause was declared by 15% of women, 53% already had at least one child, and 23% stated current motherhood desire. Notably, less than 5% of women reported to use contraceptive methods other than condoms, such as oral hormones, intrauterine device or diaphragm. The majority of women (81%) declared a stable partnership: 38% with an HIV-negative and 43% with an HIV-positive person. An occasional sexual partner was reported by the remaining 19% of cases. Among the 194 (57%) women engaged in a stable HIV-negative or an occasional partnership, 51% reported fear of infecting the partner. Nonetheless, 43% did not disclose her HIV-positive status.

**Table 1:** Characteristics of the 343 women, shown by consistency of condom use

	Total *n*=343 (100%)	Condom use “at times”/”never” *n*=152 (44%)	Condom use “always” *n*=191 (56%)	*p*
**Socio-demographic variables**				
Age, years median (IQR)	43 (37–47)	43 (36–48)	43 (38–46)	0.65*
Migrant status, *n* (%)	45 (13)	18 (12)	27 (14)	0.53§
Salary<350 €/month, *n* (%)	71 (21)	40 (26)	31 (16)	0.02*
Low education, *n* (%)	133 (39)	71 (47)	62 (33)	0.007*
Active or previous intravenous drug use, *n* (%)	73 (21)	32 (21)	41 (22)	0.93*
**HIV-related variables**				
Years from HIV diagnosis, median (IQR)	12 (6–19)	11 (6–19)	14 (7–20)	0.08§
CDC group C, *n* (%)	64/322 (20)	28/144 (19)	36/178 (21)	0.86*
HAART, *n* (%)				
Off-HAART	32 (9)	14 (9)	18 (9)	0.07*
On-HAART with plasma virological control	270 (79)	113 (74)	157 (82)	
On-HAART without plasma virological control	41 (12)	25 (17)	16 (8)	
**Women-related variables**				
Menopause, *n* (%)	52 (15)	23 (15)	29 (15)	0.99*
Motherhood desire, *n* (%)	79 (23)	32 (21)	47 (25)	0.44*
At least one child, *n* (%)	183 (53)	93 (61)	90 (47)	0.009*
Use of other contraceptive methods, *n* (%)	16 (5)	9 (6)	7 (4)	0.33*
Low satisfaction with sexual life, *n* (%)	65 (19)	30 (20)	35 (18)	0.74*
Low satisfaction with body image, *n* (%)	52 (15)	25 (17)	27 (14)	0.55*
Depressive symptoms, *n* (%)	79 (23)	39 (26)	40 (21)	0.30*
**Partner-related variables**				
At least one partner/last year, *n* (%)	38 (11)	19 (13)	19 (10)	0.45*
Partner status, *n* (%)				<0.0001*
Occasional	64 (19)	28 (18)	36 (19)	
Stable HIV-negative	130 (38)	28 (18)	102 (53)	
Stable HIV-positive	149 (43)	96 (63)	53 (28)	
No HIV disclosure to sexual partner, *n* (%)	173 (50)	101 (67)	72 (38)	<0.0001*
Fear of infecting the sexual partner, *n* (%)	132 (39)	45 (30)	87 (46)	0.002*

The percentage is the proportion of the characteristic on the total of women included in the specific column.

* Chi-square test

§ Wilcoxon test for independent samples.

Overall, 152 women stated ICU determining a prevalence of 44%. Women with less income (*p*=0.02), with lower education (*p*=0.07), and those having at least one child (*p*=0.009) reported ICU more frequently. Time of known HIV infection tended to be shorter in women declaring ICU compared to that found in women with regular condom use (*p*=0.08). As expected, higher rates of ICU were observed in women having a stable relationship with an HIV-positive partner, than in those engaged with an HIV-negative or an occasional partner (*p*<0.0.001). Furthermore, women less afraid of HIV transmission when compared to those more concerned also used condoms on an irregular bases more often (*p*=0.002). Of note, however, women declaring a sexual partner not aware about their HIV infection were more likely to use condoms inconsistently, when compared to partnerships with declared HIV status (*p*<0.0001). When analyzing women based on their antiretroviral treatment and virological control in plasma, a tendency towards a higher consistent condom use was found in those on HAART with a virological control, while those on HAART without a virological control reported ICU more often (*p*=0.07).

**Figure 1:**
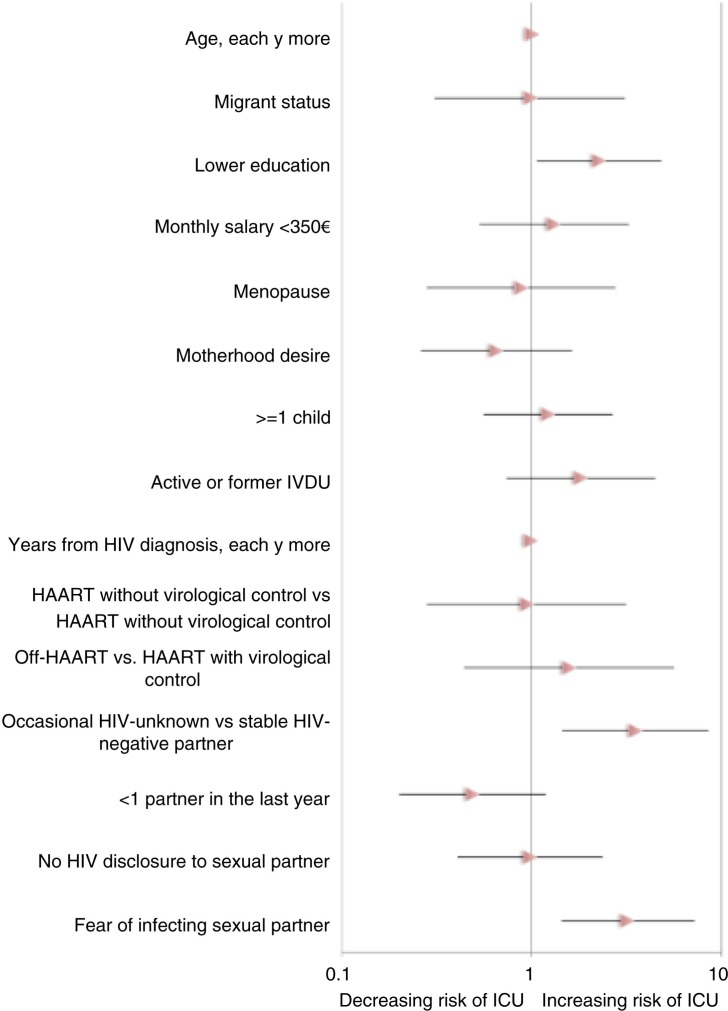
Adjusted odds ratios with 95% confidence intervals for inconsistent condom use (ICU) at multivariate analysis in the sub-group of women with an occasional HIV-unknown or stable HIV-negative partner.

In Figure 1, the results of the multivariable analysis to assess variables associated with ICU in the subgroup of women with a stable HIV-negative or an occasional HIV-unknown partner (*n*=194) are shown. Having an occasional partner (AOR 3.51, 95% confidence interval [CI] 1.44–8.54, *p*=0.005), and reporting fear of infecting the sexual partner (AOR 3.20, 95% CI 1.43–7.16, *p*=0.004) resulted in the strongest independent predictors of ICU. Current use of HAART and viraemia plasma level did not predict ICU after adjusting for demographic, behavioural and HIV-related factors. In fact, compared to women on HAART with virological control, adjusted odds ratios (AOR) of ICU were: 1.58 (95% CI 0.44–5.61; *p*=0.39) for women off-HAART and 0.95 (95% CI 0.28–3.15; *p*=0.47) for women on-HAART without virological control. Similar results were obtained when using plasma HIV-RNA 200 cp/mL as a viral load cut-off (data not shown). With regard to socio-demographic factors, lower education was the only variable significantly associated with ICU in the multivariate analysis (AOR 2.27, 95% CI 1.07–4.82, *p*=0.03).

Overall, 70% of women declared high levels of HAART adherence. The proportion of antiretroviral adherent women was 67 and 71% among those who reported ICU and consistent condom use, respectively (Chi-square *p*=0.67). No association was found between high adherence to HAART and ICU after adjusting for potential confounders (AOR 0.89, 95% CI 0.39–2.01, *p*=0.78).

## Discussion

In this cross-sectional survey carried out in Italy from November 2010 to February 2011, more than 40% of interviewed women living with HIV reported ICU and less than 5% reported the use of contraceptive methods other than condoms.

If considering condoms a primarily infection prevention measure, we were not surprised to observe that a relevant part of the women reporting ICU also declared a stable relationship with an HIV sero-concordant partner. In agreement with this finding, recently published data report sero-sorting by HIV-infected persons and increasing trends of unprotected sex in sero-concordant partnerships [14,15]. Additionally, high rates of unexpected pregnancies in the female HIV-positive population have been observed [9].

Yet our study documented that a not negligible proportion of HIV-positive females participating in the study (approximately 30%) reported ICU when engaged with a stable HIV-negative or an occasional HIV-unknown partner. Remarkably, when excluding sero-concordant couples from the analysis, no association between consistency of condom use and HAART intake together with virological control in plasma could be found. The same was observed when analyzing the association of ICU with HAART adherence. It might be therefore postulated, that awareness regarding the concept of “treatment as prevention” is still limited among women living with HIV in Italy, or at least it was until the near past.

Lower education level was a predictive factor for ICU in our study population, underscoring possibly less health information particularly in women with poor access to information sources different from the caring physician, such as Internet, patient advocacy groups, community networks and others. A recent French survey revealed a significant lack of information on prevention strategies alternative to condom use, and this was particularly true for the medical setting [4]. However, a report from Switzerland, where medical doctors presumably are highly sensitized, revealed that people living with HIV were more likely to report unprotected sex with stable partners if they were receiving antiretroviral therapy, if HIV replication was suppressed, and after the publication of the “Swiss Statement” [16]. Others observed similar results in the very last period [6,17,18].

Because of the cross-sectional nature of the study, we cannot draw any conclusions regarding the possibility of sexual risk compensation secondary to current HAART use and virological suppression in our cohort. Many authors recently argued against significant behavioural compensation after HAART initiation [5,19], even in individuals aware of the fact that efficient HAART can limit the likelihood of onward HIV transmission [4]. However, the need for interventions enhancing access to health-related information is urgent, even if the goal is different from promoting changes in sexual behaviours. In particular, being aware of the preventive role of effective HAART might facilitate HIV-positive individuals to feel less fear of transmitting HIV, to talk about onward transmission risk with sexual partners [4], and in the end to lessen HIV stigmatization. In fact, in our study population of mostly Italian women, to report fear of infecting the sexual partner was strongly associated with ICU. The direction of this association, however, could not be elucidated by our questionnaire-based study: therefore while it is possible that women having unprotected sexual intercourse with an HIV-negative or HIV-unknown partner are more scared to transmit HIV infection, it might also be possible that those not using condoms on purpose still harbours transmission distress.

Having an occasional HIV-unknown sexual partner was the other strong predictor of unprotected sexual intercourse in our study population. Some research has found that increased HIV/AIDS stigma directly or indirectly predicted unsafe sexual behaviour [20–22] and that people who perceived more HIV-related stigma were less likely to disclose their HIV-status to partners [23]. Reasons for HIV non-disclosure were not explored in this study. However, several studies have documented that disclosure of HIV infection by women may present unique risks, including fear of discrimination, rejection, violence and confidentiality concerns [9–13,24–28]. Promoting disclosure of HIV-status requires an appreciation of these many factors, and further research by behavioural and social scientists into methods for overcoming these barriers in women is needed.

Finally, looking at the findings of our study in the light of the gender power inequality, they also might reflect a male resistance to use condoms, disregarding awareness of the women's HIV sero-status. This attitude has been previously described in studies conducted both in young and older women [13,29,30] and underlines the difficulty for women with HIV to negotiate safer sex practices.

Changes in health behaviour among women are not necessarily function of their views about risk behaviour in the context of HAART. Because men are the primary users of condoms, it may be that the male partners’ view or perception regarding HIV transmission in the context of HAART will drive the change in sexual risk behaviours.

We think that the results of our study add some new information to this very concrete field of interest, even though some limitations need to be mentioned. First, the causal relationships in the significant associations could not be ascertained with our cross-sectional approach and for better understanding of the impact of specific factors on condom use, use of longitudinal data would be more appropriate. Second, when using women-reported information desirability bias cannot be completely ruled out although the questionnaire was filled anonymously and collected in sealed envelopes. The interviewed person may have had the tendency to answer the question on condom use in a manner thought to be viewed favourably by the study staff because of social desirability bias. In case this phenomenon might have occurred, prevalence of ICU among women living with HIV in Italy may even be higher than that reported, strengthening the need of studies focusing on sexual behaviour in the context of HIV. Finally, no specific information regarding the women's beliefs that may have influenced risk behaviour and specific reasons for HIV nondisclosure were assessed.

## Conclusions

Currently in Italy, use of HAART together with undetectable HIV RNA in plasma as well as antiretroviral adherence is not associated with a specific condom use pattern in women living with HIV and engaged with a sero-discordant or an HIV-unknown partner. This might suggest that the awareness of the protective role of antiretroviral treatment on HIV sexual transmission is still limited among HIV-infected persons, at least in this country. In our population of mostly Italian women, unprotected sex was significantly more frequent with occasional partners and in cases reporting fear of infecting the sexual partner, underscoring the possible role of HIV stigma and the difficulty in negotiating condom use by the female partner. Interventions enhancing access to health-related information are strongly needed in order to support the HIV-positive person in disclosure to the sexual partner. Empowerment of the female patient population to build a safe, vital and satisfactory sexual life is mandatory.

## Appendix

**DIDI Study Group**

**Study Coordinators:**

Antonella d'Arminio Monforte (Milano), Adriana Ammassari (Roma)

**Study Participants:**

Enza Anzalone (Frosinone), Teresa Bini (Milano), Antonella Castagna (Milano), Anna Maria Cattalan (Rovigo), Gabriella D'Ettorre (Roma), Fiorella Di Sora (Roma), Daniela Francisci (Perugia), Miriam Gargiulo (Napoli), Cristina Gervasoni (Milano), Nicoletta Ladisa (Bari), Giuseppina Liuzzi (Roma), Cristina Mussini (Modena), Tiziana Quirino (Busto Arsizio), Raffaella Rosso, Anna Alessandrini (Genova), Maria Paola Trotta (Roma), Francesca Vichi (Firenze)

**Experts:**

Antonella Cingolani (Roma), Rita Murri (Roma)

Statistician and Data Manager:

Paola Cicconi (Milano), Paola Pierro (Roma)

## References

[1] Vernazza P, Hirschel B, Bernasconi E, Flepp M (2008). Les personne séropositives ne souffrant d'aucune autre MST et suivant un traitement antirétroviral efficace ne transmettent pas le VIH par voie sexuelle. Bulletin des Médecins Suisses.

[2] Cohen MS, Chen YQ, Mc Cauley M, Gamble T, Hosseinipour MC, Kumarasamy N (2011). Prevention of HIV-1 infection with early antiretroviral therapy. N Engl J Med.

[3] Loutfy MR, Wu W, Letchumanan M, Bondy L, Antoniou T, Margolese S (2013). Systematic review of HIV transmission between heterosexual serodiscordant couples where the HIV-positive partner is fully suppressed on antiretroviral therapy. PLoS One.

[4] Rojas Castro D, Fugon L, Bourgeois-Fisson E, Le Gall JM, Barbier F, Spire B (2012). The “Swiss Statement”: who knows about it? How do they know? What are the effects on people living with HIV/AIDS?. AIDS Care.

[5] Fu TC, Westergaard RP, Lau B, Celentano DD, Vlahov D, Metha SH (2012). Changes in sexual and drug-related risk behavior following antiretroviral therapy initiating among HIV-infected injection drug users. AIDS.

[6] Lampe F, Speakman A, Phillips A, Sherr L, Gilson R, Johnson M (2012). ART use, viral suppression, and sexual behavior among HIV-diagnosed MSM in the UK: results from the Antiretrovirals, Sexual Transmission Risk and Attitudes (ASTRA) Study. Abstract O323; Abstract of the Eleventh International Congress on Drug Therapy in HIV Infection. J Int AIDS Soc.

[7] Orner PJ, de Bruyn M, Barbosa RM, Boonstra H, Gatsi-Mallet J, Cooper DD (2011). Access to safe abortion: building choices for women living with HIV and AIDS. J Int AIDS Soc.

[8] Ammassari A, Cicconi P, Ladisa N, Di Sora F, Bini T, Trotta M (2013). Induced first abortion rates before and after HIV diagnosis: results of an Italian self-administered questionnaire survey carried out in 585 women living with HIV. HIV Med.

[9] Raiford JL, Wingood GM, DiClemente RJ (2007). Correlates of consistent condom use among HIV-positive African American women. Women Health.

[10] Dworkin SL, Ehrhardt AA (2007). Going beyond “ABC” to include “GEM”: critical reflections on progress in the HIV/AIDS epidemic. Am J Public Health.

[11] Clum GA, Chung SE, Ellen JM, Perez LV, Murphy DA, Harper GW (2012). Victimization and sexual risk behavior in young, HIV positive women: exploration of mediators. AIDS Behav.

[12] Carrieri MP, Rey D, Serraino D, Trémolières F, Méchali D, Moatti JP (2006). Oral contraception and unprotected sex with occasional partners of women HIV-infected through injection drug use. AIDS Care.

[13] Van Devanter N, Duncan A, Birnbaum J, Burrell-Piggott T, Siegel K (2011). Gender power inequality and continued sexual risk behavior among racial/ethnic minority adolescent and young adult women living with HIV. J AIDS Clin Res.

[14] Liu C, Hu H, Goparaju L, Plankey M, Bacchetti P, Weber K (2011). Sexual serosorting among women with or at risk of HIV infection. AIDS Behav.

[15] Echenique M, Illa L, Saint-Jean G, Bustamante Avellaneda V, Sanchez-Martinez M, Eisdorfer C (2013). Impact of a secondary prevention intervention among HIV-positive older women. AIDS Care.

[16] Hasse B, Ledergerber B, Hirschel B, Vernazza P, Glass TR, Jeannin A (2010). Frequency and determinants of unprotected sex among HIV-infected persons: the Swiss HIV cohort study. Clin Infect Dis.

[17] Rodger A, Bruun T, Vernazza P, Collins S, Estrada V, van Lunzen J Understanding why serodifferent couples do not always use condoms when the HIV partner is on ART.

[18] Ayiga N (2012). Rates and predictors of consistent condom-use by people living with HIV/AIDS on antiretroviral treatment in Uganda. J Health Popul Nutr.

[19] Venkatesh KK, Flanigan P, Mayer KH (2011). Is expanded HIV treatment preventing new infections? Impact of antiretroviral therapy on sexual risk behaviors in the developing world. AIDS.

[20] Peretti-Watel P, Spire B, Obadia Y, Moatti JP (2007). Discriminations against HIV-infected people and the spread of HIV: some evidence from France. PLoS One.

[21] Wolitski RJ, Pals SL, Kidder DP, Courtenay-Quirk C, Holtgrave DR (2009). The effects of HIV stigma on health, disclosure of HIV status, and risk behavior of homeless and unstably housed persons. AIDS Behav.

[22] Varni SE, Miller CT, Solomon SE (2012). Sexual behavior as a function of stigma and coping with stigma among people with HIV/AIDS in rural New England. AIDS Behav.

[23] Przybyla SM, Golin CE, Widman L, Grodensky CA, Earp JA, Suchindran C (2013). Serostatus disclosure to sexual partners among people living with HIV: examining the roles of partner characteristics and stigma. AIDS Care.

[24] Moneyham L, Seals B, Demi A, Sowell R, Cohen L, Guillory (1996). Experiences of disclosure in women infected with HIV. Health Care Women Int.

[25] Kalichman SC, Rompa D, Cage M, Di Fonzo K, Simpson D, Austin J (2001). Initial development of scales to assess self-efficacy for disclosing HIV status and negotiating safer sex in HIV-positive persons. AIDS and Behavior.

[26] Kanuha VK, Mueller CW, Sullivan KM, Glancey P, Matsumoto P, Martel LD (2003). HIV and women in Hawaii: risk and protective factors in HIV/AIDS prevention. Hawaii Med J.

[27] Deribe K, Woldemichael K, Bernard N, Yakob B (2009). Gender difference in HIV status disclosure among HIV positive service users. East Afr J Public Health.

[28] Sullivan K, Voss J, Li D (2010). Female disclosure of HIV-positive serostatus to sex partners: a two-city study. Women Health.

[29] Neundorfer MM, Harris PB, Britton PJ, Lynch DA (2005). HIV risk factors for midlife and older women. The Gerontologist.

[30] Lindau ST, Leitsch SA, Lundberg KL, Jerome J (2006). Older women's attitudes, behavior, and communication about sex and HIV: a community-based study. J Womens Health.

